# Preoperative Psoas Muscle Index and Psoas Attenuation in Patients Undergoing Nephrectomy for Renal Cell Carcinoma: A Retrospective Cohort Study

**DOI:** 10.3390/medicina62061155

**Published:** 2026-06-14

**Authors:** Osman Murat Ipek, Erdinc Dincer, Omer Aydiner, Ahmet Halil Sevinc, Burcu Hanci Sevinc, Efe Aras

**Affiliations:** 1 Department of Urology, Kartal Dr. Lutfi Kirdar City Hospital, 34865 Istanbul, Turkey; erdinc.dincer1@saglik.gov.tr (E.D.); ahmethalil.sevinc@saglik.gov.tr (A.H.S.); burcu.hanci@saglik.gov.tr (B.H.S.); efe.aras@saglik.gov.tr (E.A.); 2 Department of Radiology, Kartal Dr. Lutfi Kirdar City Hospital, 34865 Istanbul, Turkey; omeraydiner@saglik.gov.tr

**Keywords:** renal cell carcinoma, sarcopenia, myosteatosis, psoas muscle index, prognosis

## Abstract

*Background and Objectives:* Evidence linking sarcopenia and myosteatosis to oncological outcomes in renal cell carcinoma (RCC) remains inconsistent. We aimed to evaluate whether preoperative psoas muscle measurements are associated with tumor stage, recurrence, and survival in RCC patients undergoing nephrectomy. *Materials and Methods:* A total of 199 patients who underwent nephrectomy with a diagnosis of RCC between 2018 and 2024 were retrospectively evaluated. Preoperative computed tomography images were used to measure the bilateral psoas muscle area at the L3 vertebral level and Hounsfield unit (HU) values. The psoas muscle index (PMI) was calculated. PMI and psoas HU values were analyzed as continuous variables, as this approach preserves statistical information and avoids misclassification bias inherent to arbitrary dichotomization, particularly given the absence of population-specific validated thresholds for Turkish RCC patients. Tumor stage, metastasis, recurrence, and survival data were analyzed. *Results:* The mean age of the patients was 60.29 ± 11.62 years, and 67.84% were male. The mean PMI was 5.11 ± 1.54 cm^2^/m^2^ while the mean psoas HU value was 38.63 ± 8.95 HU. PMI was significantly higher in patients with advanced-stage (T3 and T4) disease than in those with early-stage (T1 and T2) disease (5.57 ± 1.49 vs. 4.30 ± 1.28, *p* < 0.001). A positive correlation was found between T stage and PMI (r = 0.396, *p* < 0.001) and between tumor size and PMI (r = 0.215, *p* = 0.002). Modest but significant correlations were found between age and both PMI (r = −0.274, *p* < 0.001) and psoas HU values (r = −0.347, *p* < 0.001). *Conclusions:* In this retrospective nephrectomy series, conducted in a cohort inherently enriched for adequate performance status and surgical fitness, PMI showed a positive correlation with pathological T stage and tumor size, intriguingly higher PMI values were observed in patients with advanced T stages. These findings suggest that the role of psoas-based muscle measurements in the prognostication of this subset of RCC patients (advanced disease but candidates for surgical treatment) may be limited.

## 1. Introduction

Renal cell carcinoma (RCC) is a malignancy of the kidney that causes considerable mortality and has demonstrated a steady rise in incidence throughout the world [1,2]. In cases diagnosed at an early stage, partial or radical nephrectomy is preferred for curative treatment; however, unfortunately, most cases are diagnosed at later stages, where systemic spread has already occurred, resulting in a patient population in whom multiple systemic impacts can be observed at or shortly after diagnosis [1,3]. One such impact is a condition identified as cancer cachexia, which is a multifactorial syndrome characterized by progressive loss of skeletal muscle mass that cannot be reversed with conventional methods [4]. The main components of this syndrome include sarcopenia, which describes a decrease in muscle mass, and myosteatosis, which refers to the deterioration of muscle quality due to fat infiltration. These two aspects of cancer cachexia can be assessed by computed tomography (CT) [5,6].

Across multiple solid tumor types, low skeletal muscle mass and low muscle density have been associated with shorter survival, increased surgical morbidity, and reduced treatment tolerability [5,6,7]. While the sarcopenia–prognosis relationship has been investigated extensively in gastrointestinal cancers, the assessment of myosteatosis—which captures qualitative muscle deterioration through fat infiltration—remains comparatively underexplored, particularly in urological malignancies [5,6,7].

Although data on the relationship between body composition and oncological outcomes in RCC have increased in recent years, the current body of evidence shows marked heterogeneity and lacks complete consistency [1,3,8]. Studies conducted in cohorts undergoing nephrectomy for localized disease and in metastatic cases have shown that sarcopenia defined by CT may be associated with shorter overall and cancer-specific survival, earlier recurrence, and poorer oncological outcomes [3,8]. Despite these valuable results, systemic variations in analysis and definitions (some of which are unavoidable) have limited the utilization of muscle mass and quality in the prognostication of RCC. These variations include the use of different measures for muscle assessment (e.g., psoas muscle index—PMI, total skeletal muscle index, and muscle radiodensity), variability in the cutoff values for these measures, and the fact that studies have been conducted in different clinical settings [3,6,7,8].

We aimed to broadly evaluate and quantify the presence of sarcopenia (assessed by PMI) and myosteatosis (assessed by psoas muscle, Hounsfield unit—HU) in patients undergoing nephrectomy for RCC. Given the comparative scarcity of myosteatosis-focused data in surgically managed RCC, psoas HU measurements were considered the more novel of the two parameters under investigation. We also sought to assess whether sarcopenia and myosteatosis findings could be linked to tumor stage, metastasis, lymph node invasion, tumor size, recurrence, overall survival, and PFS.

## 2. Materials and Methods

### 2.1. Setting, Patients and Data Collection

This retrospective cohort study included patients who underwent radical or partial nephrectomy for RCC diagnosis at the Kartal Dr. Lutfi Kirdar City Hospital Urology Clinic between February 2018 and February 2024. The study protocol was approved by the Clinical Research Ethics Committee of Kartal Dr. Lütfi Kırdar City Hospital (Approval date: 26 March 2025, decision No:2025/010.99/14/11) and was conducted in accordance with the principles of the Declaration of Helsinki. Informed consent was neither required nor obtainable owing to the retrospective design of the study.

#### 2.1.1. Inclusion and Exclusion Criteria

**Figure 1:**
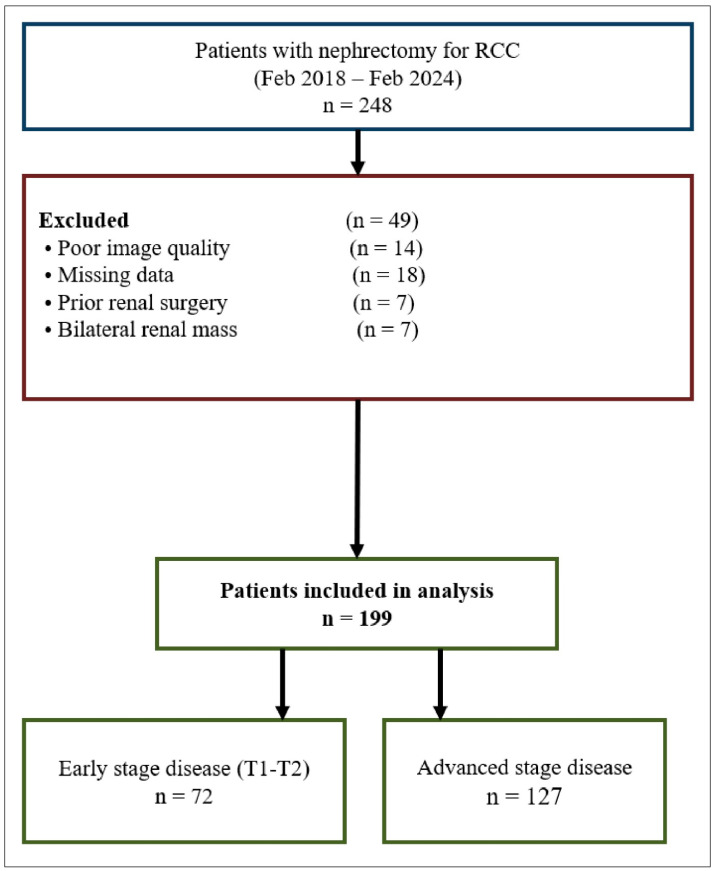
Patient selection flow chart. Of 248 patients who underwent nephrectomy for RCC between February 2018 and February 2024, 49 were excluded (poor image quality, *n* = 14; missing clinical or pathological data, *n* = 18; prior renal surgery, *n* = 10; bilateral renal masses, *n* = 7). The remaining 199 patients were included in the primary analysis and stratified into early-stage (T1–T2, *n* = 72) and advanced-stage (T3–T4, *n* = 127) subgroups for comparative analyses.

Inclusion criteria: (1) being 18 years of age or older, (2) having a histopathological diagnosis of RCC, (3) having contrast-enhanced abdominal CT imaging available in the preoperative period, and (4) having a follow-up period of at least six months. Exclusion criteria: (1) poor image quality, (2) missing clinical or pathological data, (3) previous renal surgery, and (4) bilateral renal masses. A patient selection flowchart is provided in Figure 1.

#### 2.1.2. Clinical and Pathological Data

Patient demographics (age, sex, body weight, height), comorbidities (diabetes mellitus, hypertension, coronary artery disease, chronic obstructive pulmonary disease, thyroid disorders, chronic kidney failure, other malignancies), Charlson comorbidity index, preoperative and postoperative laboratory values (hemoglobin, creatinine), surgical characteristics (surgical method, operation duration, blood loss, complications), pathological characteristics (histological type, tumor size, TNM stage, surgical margin status), and follow-up data (recurrence, metastasis, survival) were retrospectively collected from the hospital information system and patient files. Complications were categorized according to the Clavien–Dindo classification.

#### 2.1.3. Imaging Analysis and Muscle Measurements

Preoperative contrast-enhanced abdominal CT (Siemens Somatom Definition AS, Munich, Germany; 1-mm slice thickness) images of all patients were retrospectively evaluated. All measurements were performed on portal venous phase images. Psoas muscle boundaries were manually segmented on axial CT sections using the institutional PACS workstation measurement tools, with Hounsfield unit thresholding set to −29 to +150 HU to isolate muscle tissue. Psoas muscle measurements were performed at the level of the third lumbar vertebra (L3) on axial sections. Using these axial CT sections, the bilateral psoas muscle cross-sectional area (cm^2^) and mean HU values were measured. All measurements were performed by an experienced radiologist who was blinded to the patient data.

PMI was calculated by dividing the total psoas muscle area (right + left) by the square of the patient’s height (cm^2^/m^2^). The psoas HU value was determined as the arithmetic mean of the HU values obtained from the right and left psoas muscles.

### 2.2. Follow-Up Protocol

Patients were examined throughout the pre- and post-operative periods at regular intervals. Post-operative follow-ups were scheduled every 3 months during the first year, every 6 months during the second year, and a single yearly follow-up thereon. At each visit, routine laboratory tests were ordered and imaging studies were performed. Recurrence was defined as local re-emergence of disease or the detection of distant metastasis. Overall survival (OS) duration was calculated beginning from the operation date to the date of death from any cause (in cases with mortality) or the last follow-up. Progression-free survival (PFS) was also calculated from the operation date and was counted until detection of disease progression or death.

### 2.3. Statistical Analysis

All data were entered into an SPSS version 25.0 (IBM Corp., Armonk, NY, USA) dataset and analyzed in the same software (version 27, IBM, Armonk, NY, USA). Statistical significance was defined as a *p* value of less than 0.05. The conformity of the data to a normal distribution was assessed by means of histogram and Q-Q plot examination, with the Shapiro–Wilk test utilized to exclude normality assumption when necessary. Categorical variables were summarized with column frequencies and respective proportions. Descriptives for continuous data were reported as mean ± standard deviation if each group met normal distribution assumptions and as median (Q1–Q3 quartiles, IQR) for those without normal distribution. PMI and psoas HU were analyzed as continuous variables in all primary analyses, as this approach preserves statistical power and avoids misclassification bias associated with arbitrary dichotomization. Analyses of psoas measurements were performed using Student’s *t*-test due to the normality of distribution. Relationships between psoas measurements and other variables were evaluated using Pearson’s or Spearman’s correlation coefficients, depending on the normality of distribution.

## 3. Results

A total of 199 RCC patients were included in the study (Figure 1). The mean age of the patients was 60.29 ± 11.62 years, and 67.84% were male (*n* = 135). The median Charlson comorbidity index was 5 (IQR: 3–6). At the time of diagnosis, 23 patients (11.56%) had metastases. Among these, 12 (6.03%) had lymph node metastases (LAP), 8 (4.02%) had distant organ metastases, and 3 (1.51%) had both. Laparoscopic nephrectomy was performed in 82.41% of patients (*n* = 164), while conversion to open surgery was required in 19 patients (9.55%). Pathological examination revealed clear cell carcinoma in 73.87% of patients (*n* = 147), and the median tumor size was 65 mm (IQR: 45–90 mm). The majority of patients were classified as stage T3a (58.79%, *n* = 117). The high proportion of T3a disease likely reflects the referral and case-mix pattern of a tertiary urology center and may not be representative of the general RCC surgical population. The median follow-up period was 36 months (IQR: 22–55), during which recurrence developed in 9 patients (4.52%) and lymph node invasion developed in 7 patients (3.52%). In muscle measurements, the mean PMI was 5.11 ± 1.54 cm^2^/m^2^ and the mean psoas HU was 38.63 ± 8.95 HU (Supplementary Table S1).

**Figure 2:**
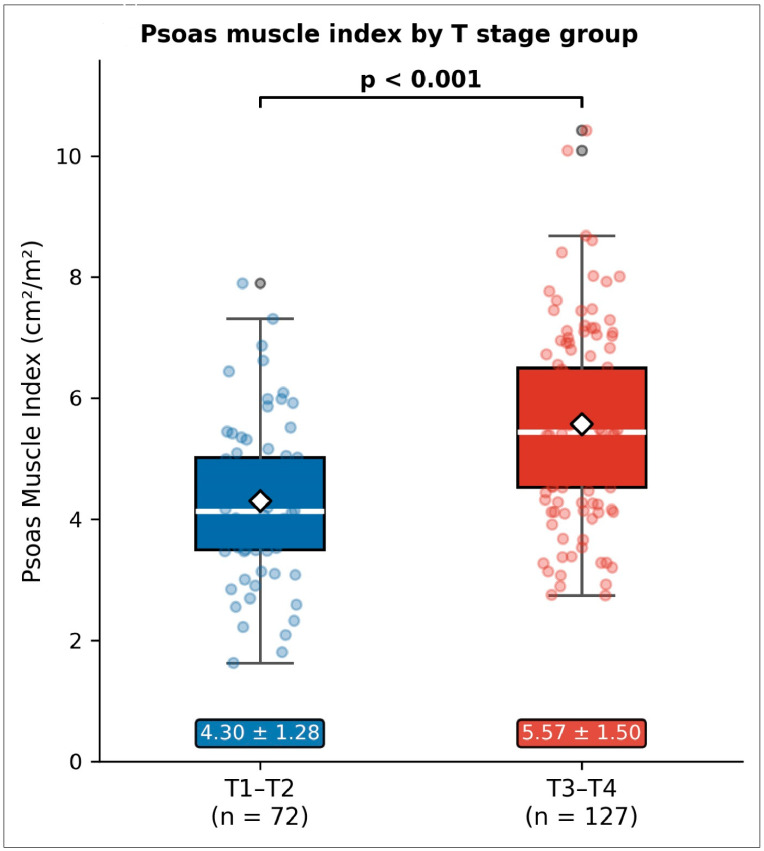
Box-and-whisker plots comparing psoas muscle index (PMI) between early-stage (T1–T2) and advanced-stage (T3–T4) renal cell carcinoma patients. Individual data points are overlaid as jittered dots. Diamonds indicate group means ± standard deviation. PMI was significantly higher in the T3–T4 group (5.57 ± 1.49 vs. 4.30 ± 1.28 cm^2^/m^2^; *p* < 0.001).

When evaluating the relationship between psoas muscle measurements and patient and tumor characteristics, the most significant difference in PMI was detected according to T stage. The PMI (5.57 ± 1.49 cm^2^/m^2^) was significantly higher in advanced-stage (T3 and T4) patients compared to early-stage (T1 and T2) patients (4.30 ± 1.28 cm^2^/m^2^) (*p* < 0.001), as illustrated in Figure 2. To contextualize this discrepancy, among T1–T2 patients, 61.1% had a PMI above the cohort median (5.04 cm^2^/m^2^), compared to 55.6% in the T3–T4 group, indicating that the higher mean PMI in the advanced-stage group was driven by a subset of large-built patients with high-volume tumors. When psoas muscle HU values were examined, we found that they were significantly higher in patients with other malignancies (43.71 ± 5.62 HU) compared to those without (38.30 ± 9.03 HU) (*p* = 0.042). No significant relationships were found with other clinical or pathological parameters (Table 1).

**Figure 3:**
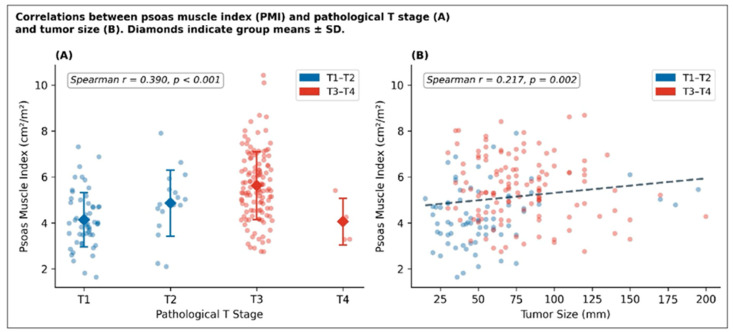
Scatterplots illustrating correlations between psoas muscle index (PMI) and (**A**) pathological T stage and (**B**) tumor size (mm). Data points are color-coded by T stage group (blue: T1–T2; red: T3–T4). Diamonds indicate group means ± SD in panel A. A dashed regression line is shown in panel B. Moderate positive correlation: PMI vs. T stage (Spearman r = 0.396, *p* < 0.001); weak positive correlation: PMI vs. tumor size (Spearman r = 0.217, *p* = 0.002).

The results of the correlation analysis between psoas muscle measurements and patient and tumor characteristics are presented in Table 2 and Figure 3. A weak negative correlation was found between PMI and age (r = −0.274, *p* < 0.001). A weak positive correlation was found between tumor size and PMI (r = 0.215, *p* = 0.002). There was a moderate positive correlation between T stage and PMI (r = 0.396, *p* < 0.001). A weak negative correlation was found between psoas muscle HU values and age (r = −0.347, *p* < 0.001). No significant correlations were found for the Charlson comorbidity index, Clavien–Dindo complication classification, and the preoperative and postoperative hemoglobin or creatinine values (*p* > 0.05 for all).

## 4. Discussion

In this single-center, retrospective nephrectomy cohort, we evaluated preoperative CT images to evaluate PMI (at the L3 level) and HU values of the psoas muscle in patients with RCC and sought potential associations with pathological characteristics and oncological outcomes. The prominent findings of the study were that PMI was intriguingly higher in pathologically advanced tumors and that PMI was positively correlated with tumor size (Figure 2 and Figure 3). In contrast, no significant relationship was found between PMI or HU and the presence of metastasis at diagnosis, lymph node invasion, or postoperative recurrence. Similarly, in OS and PFS analyses, these muscle parameters did not emerge as independent predictors beyond established prognostic variables such as tumor stage, metastatic disease, and nodal involvement.

It has been reported that sarcopenia, defined by the skeletal muscle index (SMI) measured at the L3 level before nephrectomy in organ-confined disease, is associated with worse overall and cancer-specific survival and remains independent in multivariate analyses [9]. Corroborating evidence showing significantly shorter recurrence-free survival in cases with low psoas muscle volume and low PMI has been described [10]. In fact, reduced skeletal muscle mass after nephrectomy in localized RCC has been associated with all-cause mortality, and muscle loss has been described as an additional prognostic parameter that was independent of classic tumor variables [11,12]. Supportive evidence to this relationship has also been reported in Turkish patients, wherein a large localized RCC cohort was examined and demonstrated that sarcopenia (defined by PMI/SMI) was an independent poor prognostic factor for recurrence, shorter survival, and mortality [8]. Despite consistent evidence for the general RCC population, the higher PMI observed in patients with advanced-stage disease in our series raises several questions that might be explained by multiple factors. The most critical is surgical selection bias: this study exclusively included patients deemed fit for nephrectomy. Patients with severely depleted muscle mass and poor functional reserve—who are most likely to exhibit the canonical sarcopenia–prognosis relationship—were systematically excluded. Accordingly, the present findings should not be generalized to patients with advanced RCC managed non-surgically or with palliative intent. It is also possible that larger tumor masses are detected in larger-built individuals, which could confound the measurement of psoas area in this context [13]. Thirdly, the ‘obesity paradox’ may bias results. In advanced-stage patients receiving targeted therapy, there may exist a confounded relationship between survival and the BMI value (and adipose tissue mass). As such, measured PMI values could positively correlate with stage regardless of the direct underlying relationship between these variables—due to variations in tumor size or treatment tolerance (higher in larger/muscular patients) [14]. As formal adipose tissue measurements were not available in the current study, this interpretation remains speculative and warrants prospective investigation. Therefore, our findings suggest that psoas-based measurements in this selected surgical population may represent body composition and the threshold for surgical acceptability rather than the functional and physiological impacts of cancer cachexia and/or sarcopenia.

The significant decrease in PMI and psoas HU values with increasing age indicates that aging not only reduces muscle mass but also simultaneously impairs muscle quality. This finding is consistent with available evidence showing that sarcopenia cannot be clinically explained solely by a quantitative reduction in muscle mass [15,16,17]. Experimental and clinical data have shown that muscle attenuation measured by CT is associated with intramuscular lipid accumulation and that lower attenuation is associated with reduced muscle strength independently of muscle cross-sectional area [18,19]. The increase in myosteatosis with age suggests that these changes may be partially independent of body weight dynamics and may be associated with adverse metabolic outcomes [20]. The demonstration of negative correlations with age (for both PMI and HU) in our series clearly shows that psoas measurements are sensitive enough to capture the impact of aging on muscle mass and muscle quality. The lack of strong relationships with tumor stage or recurrence reinforces the possibility that the age-related decrease in muscle mass and quality in this particular population is likely a result of frailty due to aging, comorbidity, and performance status rather than disease-specific findings [15,16,21]. The relationships with HU indicate that outright muscle loss and fat infiltration have a concerted effect on muscle degeneration, which might be associated with multiple factors, including inflammation, oxidative burden, disease status, and aging [15,17,19,22]. However, although the psoas muscle is frequently used in practice, it has been reported that the psoas area and radiodensity may not always fully represent the entire L3 skeletal muscle compartment, causing misrepresentation of the prevalence of myosteatosis [21]. When evaluated together, the decrease in muscle mass and quality should be interpreted as a subtle indicator of vulnerability, which must be assessed in conjunction with comorbidities and on a case-by-case basis.

While the relationship between sarcopenia and RCC outcomes has been examined in prior retrospective cohorts and meta-analyses, myosteatosis—reflected in the current study by psoas HU values—remains comparatively underexplored in surgically managed RCC and represents the more novel aspect of the present investigation. Meta-analyses have shown that myosteatosis is associated with both all-cause and cancer-specific mortality in many solid tumor types, independent of skeletal muscle mass [5,23]. Low muscle in patients with many types of cancer, including colorectal, breast, liver, and lung cancers, has been associated with poor prognosis. In patients with gastrointestinal cancer undergoing surgery, myosteatosis has been shown to be associated with early postoperative complications and long-term mortality [23,24,25]. In RCC specifically, it has been reported that high muscle mass is independently associated with better survival in cohorts receiving targeted therapy among metastatic patients and that muscle mass can provide additional prognostic information [26,27]. However, in our series of patients who underwent nephrectomy for localized or locally advanced disease, no significant relationship was found between either tumor aggressiveness indicators or short- to medium-term oncological outcomes. Possible reasons include the limited representativeness of single-slice HU measurements based on the psoas muscle, the problem of standardization of HU cut-off values for myosteatosis, and uncertainty regarding the suitability of the selected thresholds for the Turkish RCC population [23]. Additionally, the limited number of recurrence and mortality events may have constrained statistical power.

Our findings should be evaluated within the context of several important limitations. The retrospective, single-center design and restriction to surgically eligible patients constitute a significant selection bias, limiting generalizability to the broader RCC population. This selection bias is the most plausible explanation for the paradoxical positive association between PMI and pathological stage and must be prominently considered when interpreting all findings in this report. Performing muscle measurements on a single L3 slice via the psoas alone may not accurately represent the entire L3 skeletal muscle compartment. Formal Kaplan–Meier and Cox regression survival analyses were not performed for two reasons: first, only 9 recurrence events were observed (4.52%), falling below the minimum of 10 events per variable generally required for reliable survival estimation; second, survival data were only available as duration data, without a binary event indicator for mortality, precluding the construction of properly censored survival curves. Presenting such analyses under these conditions would risk producing statistically unreliable results. Furthermore, population-specific validated sarcopenia and myosteatosis thresholds for Turkish RCC patients are not yet available, increasing the risk of misclassification should dichotomization be applied. The absence of functional muscle assessments such as grip strength or gait speed is a further limitation, as CT-defined muscle depletion does not always correspond to clinical functional impairment.

## 5. Conclusions

In our carefully selected cohort of patients undergoing nephrectomy for RCC—a series inherently enriched for adequate performance status and surgical fitness—PMI and psoas muscle HU values measured by preoperative CT did not show significant associations with metastasis, nodal involvement, recurrence, or short- to medium-term survival duration. The positive correlation between pathological stage and PMI suggests that the previously reported sarcopenia–prognosis relationship may not apply to patients with advanced-stage disease who remain candidates for surgical treatment, most plausibly as a consequence of the systematic exclusion of frail and sarcopenic patients from surgical cohorts. This underscores the need for fine-tuned assessment of psoas-based measurements in RCC prognostication, stratified by disease stage and surgical candidacy. The comparative scarcity of myosteatosis data in surgically managed RCC identified in this study further highlights the need for dedicated investigation of muscle quality parameters in this subset. To determine the true prognostic contribution of muscle composition parameters, measurement standardization, the definition of population- and subtype-specific thresholds, and prospective multicenter validation studies are required.

## Figures and Tables

**Table 1:** Summary of psoas measurements with regard to patient and tumor characteristics.

	Psoas Muscle Index	*p*	Psoas HU, Average	*p*
Other malignancy				
No	5.12 ± 1.56	0.842	38.30 ± 9.03	**0.042**
Yes	5.02 ± 1.29		43.71 ± 5.62	
Metastasis, preoperative				
No	5.07 ± 1.55	0.357	38.40 ± 9.18	0.328
Yes	5.39 ± 1.45		40.35 ± 6.79	
Distant metastasis, preoperative				
No	5.08 ± 1.55	0.330	38.68 ± 9.00	0.758
Yes	5.55 ± 1.31		37.82 ± 8.38	
Complication				
No	4.99 ± 1.50	0.096	38.48 ± 8.57	0.730
Yes	5.38 ± 1.61		38.96 ± 9.80	
Pathology				
Clear cell	5.13 ± 1.57	0.742	38.43 ± 9.37	0.598
Other	5.05 ± 1.46		39.19 ± 7.69	
T stage				
T1 and T2	4.30 ± 1.28	**<0.001**	37.08 ± 10.76	0.095
T3 and T4	5.57 ± 1.49		39.50 ± 7.64	
Surgical margin				
Negative	5.08 ± 1.49	0.386	38.51 ± 8.98	0.446
Positive	5.65 ± 2.18		40.54 ± 8.54	
Recurrence				
No	5.14 ± 1.55	0.281	38.79 ± 9.02	0.243
Yes	4.57 ± 1.15		35.22 ± 6.76	
Lymph node invasion				
No	5.12 ± 1.54	0.815	38.54 ± 9.07	0.490
Yes	4.98 ± 1.60		40.93 ± 4.00	

Descriptive statistics are presented using mean ± standard deviation for normally distributed continuous variables. *p* values were obtained using Student’s *t*-test. Statistically significant *p* values are shown in bold.

**Table 2:** Correlations between psoas measurements and patient and tumor characteristics.

		Psoas Muscle Index	Psoas HU, Average
Age	r	−0.274 ^†^	−0.347 ^†^
	p	**<0.001**	**<0.001**
Charlson comorbidity index	r	0.052 ^‡^	−0.035 ^‡^
	p	0.466	0.625
Clavien–Dindo classification	r	0.081 ^‡^	−0.012 ^‡^
	p	0.258	0.868
Hemoglobin, Preoperative	r	−0.133 ^†^	−0.051 ^†^
	p	0.060	0.472
Hemoglobin, Postoperative	r	−0.073 ^†^	0.000 ^†^
	p	0.303	0.996
Creatinine, Preoperative	r	0.005 ^‡^	0.010 ^‡^
	p	0.942	0.892
Creatinine, Postoperative	r	0.072 ^‡^	0.066 ^‡^
	p	0.314	0.354
Tumor size, mm	r	0.215 ^‡^	−0.059 ^‡^
	p	**0.002**	0.405
T stage	r	0.396 ^‡^	0.071 ^‡^
	p	**<0.001**	0.320

^†^ Pearson correlation coefficient, ^‡^ Spearman correlation coefficient. Statistically significant *p* values are shown in bold.

## Data Availability

The data presented in this study are available on request from the corresponding author.

## Supplementary Materials

The following supporting information can be downloaded at https://www.mdpi.com/article/10.3390/medicina62061155/s1, Table S1. Summary of patient and tumor characteristics and psoas measurements.

## Abbreviations

BMI	body mass index
CT	computed tomography
HU	Hounsfield unit
IQR	interquartile range
L3	third lumbar vertebra
LAP	lymphadenopathy
OS	overall survival
PFS	progression-free survival
PMI	psoas muscle index
RCC	renal cell carcinoma
SMI	skeletal muscle index
TNM	tumor, node, metastasis
