# Supplementary material for: Preoperative Psoas Muscle Index and Psoas Attenuation in Patients Undergoing Nephrectomy for Renal Cell Carcinoma: A Retrospective Cohort Study
Source: Medicina (Kaunas). 2026 Jun 14;62(6):1155. doi: 10.3390/medicina62061155 (PMC13303003; doi:10.3390/medicina62061155)
Supplement: Supplementary file 1 [file medicina-62-01155-s001.zip › medicina-4295529-supplementary.pdf]

Table S1. Summary of patient and tumor characteristics and psoas measurements.

Age	60.29 ± 11.62
Sex	
Female	64 (32.16%)
Male	135 (67.84%)
Height, m	1.72 (1.62 - 1.76)
Diabetes mellitus	49 (24.62%)
Hypertension	80 (40.20%)
Coronary artery disease	32 (16.08%)
Chronic obstructive pulmonary disease	19 (9.55%)
Thyroid diseases	13 (6.53%)
Chronic renal failure	7 (3.52%)
Other malignancy	12 (6.03%)
Charlson comorbidity index	5 (3 - 6)
Side	
Right	110 (55.28%)
Left	89 (44.72%)
Metastasis, preoperative	23 (11.56%)
LAP	12 (6.03%)
Distant	8 (4.02%)
Both	3 (1.51%)
Operation time, min	120 (120 - 160)
Type of operation	
Laparoscopic	164 (82.41%)
Open	16 (8.04%)
Conversion from laparoscopic to open	19 (9.55%)
Reason of conversion	
Cohesion	8 (42.11%)
Hemorrhage	4 (21.05%)
Thrombus	4 (21.05%)
Local invasion	3 (15.79%)
Amount of bleeding, mL	300 (100 - 800)
Complication (1)	61 (30.65%)
Gastrointestinal tract	4 (2.01%)
Infection	15 (7.54%)
Genitourinary	3 (1.51%)
Hematologic/Vascular	38 (19.10%)
Cardiac	0 (0.00%)
Wound/Skin	1 (0.50%)
Pulmonary	11 (5.53%)
Neurological	1 (0.50%)
Metabolic	5 (2.51%)
Clavien-Dindo classification	
Grade 0	138 (69.35%)
Grade 1	9 (4.52%)
Grade 2	43 (21.61%)
Grade 3	7 (3.52%)
Grade 4	2 (1.01%)
Hemoglobin	
Preoperative	13.13 ± 2.00
Postoperative	11.82 ± 1.92

Creatinine	
Preoperative	0.90 (0.75 - 1.07)
Postoperative	1.20 (1.03 - 1.45)
Pathology	
Clear cell	147 (73.87%)
Papillary	23 (11.56%)
Chromophobe	18 (9.05%)
Other	11 (5.53%)
Tumor size, mm	65 (45 - 90)
T stage	
T1a	26 (13.07%)
T1b	29 (14.57%)
T2a	12 (6.03%)
T2b	5 (2.51%)
T3a	117 (58.79%)
T3b	6 (3.02%)
T3c	0 (0.00%)
T4	4 (2.01%)
Positive surgical margin	12 (6.03%)
Recurrence	9 (4.52%)
Lymph node invasion	7 (3.52%)
Follow-up time, months	36 (22 - 55)
Psoas area, cm^2	
Right	7.36 ± 2.64
Left	7.58 ± 2.79
Total	14.94 ± 5.30
Psoas muscle index	5.11 ± 1.54
Psoas HU	
Right	38.49 ± 9.24
Left	38.76 ± 9.41
Average	38.63 ± 8.95

Descriptive statistics are presented using mean ± standard deviation for normally distributed continuous variables, median (25th percentile - 75th percentile) for non-normally distributed continuous variables and frequency (percentage) for categorical variables. (1) Patients may have more than one of the following.
